# Addressing the Triple Trauma of Factors Leading to Perinatal Health and Mental Health Consequences in Two Upstate New York Communities

**DOI:** 10.3390/bs15010020

**Published:** 2024-12-29

**Authors:** Robert H. Keefe, Robert A. Rubinstein, Kiara Van Brackle, Sanid Music, Zikora Nnam, Sandra D. Lane

**Affiliations:** 1 School of Social Work, University at Buffalo, Buffalo, NY 14260, USA; 2 Department of Anthropology, Maxwell School of Citizenship and Public Affairs, Syracuse University, Syracuse, NY 13244, USA; rar@syr.edu; 3 Department of Sociology, Maxwell School of Citizenship and Public Affairs, Syracuse University, Syracuse, NY 13244, USA; kavanbra@syr.edu; 4 Liverpool High School, Liverpool, NY 13090, USA; musicsanid07@gmail.com; 5 Department of Public Health, Syracuse University, Syracuse, NY 13244, USA; zinnam@syr.edu; 6 Public Health & Anthropology, Syracuse University, Syracuse, NY 13244, USA; sdlane@syr.edu

**Keywords:** neighborhood health, maternal and child health, family health, interpersonal violence, perinatal mood disorders, father involvement, racially disproportionate incarceration

## Abstract

This article focuses on the impact of trauma experienced by individuals, families and groups, and neighborhoods in Rochester and Syracuse, New York. Using the levels of analysis put forward in Bronfenbrenner’s ecological systems theory (i.e., individual, family, and community), we argue that trauma operates at each of those levels. This mixed-methods study reviews the findings of seven previously published studies (with data collection ranging from 2000 to 2019), each of which addresses trauma among low-income residents. Specific methods include secondary analyses of births, qualitative interviews of persons who delivered a baby within the past two years, a community survey of residents living in high-crime areas, and secondary birth data to document the impact of socio-ecological risk factors on the trauma of birthing persons, their children, and their partners. Individuals and families living in high-risk neighborhoods (e.g., where residents experience frequent gun shots, racially disproportionate incarceration, and poverty) had more negative health outcomes including posttraumatic stress disorder, intrauterine growth restriction, and depression. Interventions focused on community-based practices that address individual, family, and community-level trauma must also address the multiple risk factors for trauma located in the environmental and social stressors.

## 1. Introduction

In this article, we apply the concept of trauma to the analysis of health and mental health disparities of low-income parents with infants and young children living in Rochester and Syracuse, New York. The American Psychological Association defines trauma as a discrete or ongoing event that results in intense fear and has an ongoing negative effect on an individual’s ability to function.

This article synthesizes previous studies conducted by our research team in Rochester and Syracuse, NY, from 2000 to 2019. Each of those studies focused on pregnancy and children in families coping with stressors in their environment. Integrating those studies in this analysis of trauma, illustrates its multifaceted etiology. The studies are supplemented with recent publicly available data. We use Bronfenbrenner’s (1983, 1994) ecological system’s theory to integrate several environmental and social health risks to provide a more complete and complex description of the health and mental health consequences of trauma during childbirth and family formation.

## 2. Literature Review

Bronfenbrenner’s ecological systems theory brings together the biological, psychological, and social sciences to explain the ecology of human development (Crawford, 2020). Bronfenbrenner argued that individuals interact with others in a dynamic and interconnecting system of individuals, families, groups, and communities (Bronfenbrenner, 1983). These interacting systems are in turn affected by local, state, and federal health and social policies that influence an individual’s life experiences over the course of time (Bronfenbrenner, 1994).

Individuals living in high-risk communities often face traumatic life experiences that affect not only themselves but also their families, neighborhoods, and communities as well. Traumatic events in turn become ingrained within the individual’s intersecting family and community networks (Fitzgerald et al., 2021) to the point that traumatic events become normalized (Lane et al., 2017). Various researchers argue that trauma is inescapable and needs to be considered in all clinical settings (Kessler et al., 1995). Trauma leads to ongoing risk for developing additional psychiatric illnesses such as posttraumatic stress disorder (PTSD), a disorder resulting from imagined or actual threats to a person’s life or welfare resulting in distressing thoughts, feelings, or dreams associated with the threats and efforts to avoid cues associated with the trauma (APA, 2024) and depression (Mauritz et al., 2013). Lane et al. explained how trauma resulting from neighborhood violence is rooted in the long history of urban renewal, the Rockefeller drug laws, and the de-industrialization that helped devastate communities of color (Lane et al., 2017). Community violence, clustering in densely populated neighborhoods, creates unmanageable stress for the families who live within them. Butcher et al. conclude that simply living in disorganized neighborhoods often resulted in greater exposure trauma (Butcher et al., 2015). Moreover, racial bias in incarceration results in single-parent households, greater poverty, and young people of color being at greater risk for exposure to violence and trauma (Lane et al., 2004). Elevated homicide, particularly of Black males, who themselves are often parents, renders their families vulnerable to grief, loss, and greater poverty (Smith & Patton, 2016). It also has what community members describe as a ripple effect of trauma so that each homicide affects roughly 200 other people in communities of color in part because the residents are densely settled and many know each other very well (Jennings-Bey et al., 2015).

Among birthing individuals, exposure to community-based trauma has been associated with various psychiatric disorders including posttraumatic stress disorder, which affects the development of maternal, family, and child relationships (Erickson et al., 2019; Keefe et al., 2019). Maternal depression, which often results from exposure to trauma, can also negatively affect parenting behavior and worsen childhood health outcomes and psychosocial development (Cook et al., 2018; Muzik et al., 2017; van Ee et al., 2016). Birthing individuals living with an abusive male partner frequently become isolated and cut off from others within their once more developed social-ecological network (Flecha, 2021; Keefe et al., 2021), which has been shown to adversely impact the birthing individuals’ ability to function as parents (Fusco et al., 2016; Timshel et al., 2017). Intrauterine growth restriction (IUGR) indicates a fetus whose growth is less than 10 percent on standardized fetal growth charts. Fetal growth in utero can been compromised by inadequate nutrition and severe trauma. Pregnant persons living in food deserts and neighborhoods with elevated gunshots and injuries may be affected by both of those risks (Bennet et al., 2018; Kinsella & Monk, 2009; Rocha et al., 2024).

Many of the studies on trauma during the perinatal period focus predominantly on individual traumatic experiences and the risk factors for that trauma. The current study extends the focus to socio-ecological risk factors that affect individuals, their families, and the communities in which they live.

## 3. Methods

### Research Locations

In our Rochester, NY study, the participants resided in Monroe County census tract 93.01 (n = 2554), which is characterized in the US Census as having 52.8% of families with children under the age of 18 living below the federal poverty level. In that census tract, the racial identities of the residents are 18.6% white, 38.6% African American, 42.8% other, and 31.9% of Latinx ethnicity. Our studies in Syracuse took place in various neighborhoods. In Syracuse, 45.8% of families with children under the age of 18 live below the federal poverty level, a rate that is one of the highest in the nation (Kielar, 2024). Many of the Rochester and Syracuse neighborhoods are sites of gun violence and murders, perpetrated to a large extent by adolescents and young adults involved in gang activity (Bergen-Cico et al., 2018; Garzone, 2021; Rubinstein et al., 2018). Syracuse’s population of 148,620 is composed of 41% white, 31% African American, 7% Asian, and 21% residents of other backgrounds, among whom 11% are of Latinx ethnicity. Syracuse has three refugee resettlement agencies, bringing asylum seekers from war-torn and disaster-affected parts of the world. In Syracuse, from 2017 to 2019, 23% of infants were born to foreign-born persons, the majority to those from refugee-sending countries (Goble et al., 2023). The birth countries of the foreign-born birthing persons were (in rank order, of countries represented by 10 or more births) Somalia, Burma, the Democratic Republic of the Congo, Bhutan, Cuba, Kenya, Yemen, Sudan, China, Thailand, Nepal, Iraq, and Vietnam.

Our research team examined trauma at the individual, family, and community levels, following Bronfenbrenner’s ecological systems theory, which argues that the levels are not mutually exclusive, but instead inform and influence each other. We highlight community-risk factors, such as limited access to healthy food, which negatively affects the birth weights of infants born to pregnant individuals living in those areas. Likewise, an individual child who is the victim of a drive-by shooting, may broadly affect community members who grieve over that child’s loss and fear that another child may be next. With this in mind, although some traumatic experiences (e.g., PTSD) are measured at the individual level, they are so prevalent in the community that they may also indicate the likelihood of community-level trauma. Joseph et al. (2022) argue that viewing PTSD as solely a medical diagnosis of the individual removes the community context that shapes trauma and prevents practitioners from identifying the basis of diagnostic guidelines. Consequently, the concept of trauma, they argue, should be recognized through a biopsychosocial lens and not strictly a biomedical framework.

The seven studies, which address health and mental health disparities experienced by impoverished families, have been conducted over two decades in an iterative manner. Those studies are supplemented with publicly available data. Table 1 outlines the studies used in this analysis, including the study’s topic, methods, participants, and location.

## 4. Results

Table 2 shows the individual, family, and community levels of trauma that we address, as well as the special case of refugees who have experienced trauma prior to resettlement.

*Individual level—intimate partner violence*

The individual level addresses trauma experienced by the birthing persons themselves, including intimate partner violence (IPV). A secondary analysis of prenatal and birth data evaluated the prevalence of intentional injury to the birthing person by their significant other (Leone et al., 2010). The study population of 2873 births was 40.6% white, 48.5% African American, and 10.9% of other racial backgrounds. During the initial prenatal care sessions, the pregnant individuals were asked two questions: “Have you been hit, slapped, punched, or otherwise hurt by your partner?” and “Do you feel unsafe in your home right now?” A total of 3.7% of the birthing persons responded that they were subject to this type of trauma; among white individuals the rate was 3.6% and among African Americans it was 4%. A logistic regression model found that birthing persons who reported IPV *were over 14 times more likely to experience pregnancy trauma* (i.e., diagnosed physical injury during the pregnancy). Assaults included being kicked or stabbed in the abdomen, being sexually assaulted, attempted suicide following rape, and one individual who reported being shot with a gun in the abdomen. IPV-exposed birthing persons were also *four times as likely to experience placental abruption*, a life-threatening condition in which the placenta peels away from the uterus prior to the birth, which can cause internal hemorrhage and fetal hypoxia. The two narratives below of two individuals who recently gave birth, from the Rochester qualitative study of postpartum depression describe their trauma at the hands of their partners:

I had a cut on my forehead. I had eleven stitches ‘cause I said I was leaving him. [He] [p]ressed the knife against my forehead, … and then I had a black eye. he hit me so hard on my birthday, and I passed out, [he] beat me with a broom. …

…the minute that I found out that I was pregnant, everything just changed. I was getting hit on my whole nine months that I was pregnant. I delivered her with a black eye. He beat me so bad three days before I had her, I don’t know what made me stay. I guess I just thought that maybe he would change. … but he never did. … it just got worse.

*Individual level—isolation*

For many individuals, residing in high-crime neighborhoods results in social isolation (Tung et al., 2019). In Rochester, where our team interviewed African American and Latinx persons who had given birth within the past two years, the respondents reported they wanted to have greater connection with other birthing individuals, but found they had too little time or their lives were too complicated to spend time with other people (Keefe et al., 2021). One of the Rochester participants described her experience of isolation as,

“I[t is] really more about my kids. You know, nerves. I just worry so much, ‘cause I got two [kids] in Trinidad, one [is] with my mom…. I’m trying to move back to New York [City] once my son finishes with school, and I have the baby. I be worried so much thinking that I don’t have nobody to help me. ‘Cause I don’t have family up here and where I live is not safe”.

*Family level—missing men*

In 1973 New York State passed what has become known as the “Rockefeller Drug Laws”, which mandated lengthy sentences for drug-related crimes (Keefe et al., 2017). Although we have indirect evidence that individuals of white, African American, and Latinx racial/ethnic backgrounds use illicit drugs at similar rates, African Americans have been disproportionately incarcerated under that law (Keefe et al., 2017). A 2007 study of all US counties with a population of 250,000 or greater, comparing African American and white populations sentenced to a carceral facility in 2006, found Onondaga County, which contains Syracuse, to have the second highest proportion of racially-biased, drug-related sentencing (Justice Policy Institute et al., 2007). Disproportionate incarceration affects males more than females, as demonstrated by individuals sentenced in New York State, in which 96% were male and 4% female (NYS Department of Corrections and Community Supervision, 2022). The racial bias in sentencing results in a skewed sex ratio among African Americans. In Syracuse, according to census data (2000–2020), between the ages of 25 and 29, African American women vastly outnumber African American men, whereas in the white population, the male–female ratio in those years is nearly equal (Lane et al., 2004). The missing men are often fathers and their incarceration hurts their children. In 1970, prior to the implementation of the Rockefeller drug laws, 70.4% of African American families with children under age 18 in Onondaga County were two-parent families. By 1990, 61.8% of African American families were female-headed (Lane et al., 2004). Single-parent households have greater poverty than those with two-parent incomes, forcing many of the single parents to live in rental properties in neighborhoods that are hard-hit by gunshots and other violence. Among births to unmarried partners, in which the father does not sign the Declaration of Paternity (DP) the *infant was nearly four times as likely to die between the first month of life and the first birthday* (Lane et al., 2017; Lane et al., 2004; Brandt et al., 2022). Qualitative interviews indicated that a major reason why a father would not sign the DP is that he is incarcerated.

*Family level—fear of children being killed*

In Syracuse, much of the neighborhood violence claims the lives of children, while being disproportionately perpetrated by adolescents and young adults. Nearly 25% of murder victims were children or teens and nearly 30% of the perpetrators were age 18 or younger (Hayes, 2024b). In 2021, 11-month-old Dior Harris, and two of her cousins, ages three and eight, were shot while sitting in the backseat of a car as a result of crossfire from a drive-by shooting. The children’s mothers were in the front seats of the car, with the children in the back. The mothers did not realize that their children had been hit, until one child said, “Mommy, it’s burning”, upon which the mother turned and saw the blood (Misiaszek, 2024).

In 2023, 11-year-old Brexialee Torres-Ortiz was returning home from buying a quart of milk at the corner store to make macaroni and cheese. Brexi was the president of her fifth-grade class (Pelisek, 2023). She was shot in the back and killed in a drive-by shooting. Her mother’s victim statement in court stated that she “still has not figured out how to live in a world without Brexi”. She told the court that in the morning she still finds herself looking for her daughter when she first wakes up. She said she also has to explain to her 6-year-old daughter why Brexi is not here” (Hayes, 2024a). Although her mother stated that she has not yet forgiven the perpetrators, she said that she believed that Brexi “would have forgiven them”.

*Community level—neighborhood violence and PTSD*

In 2015, we conducted a survey of adults (age 18 and older) in the Syracuse neighborhoods with elevated gunshots at the Juneteenth celebration, youth sports events, and a hair braiding salon (Lane et al., 2017). Although the survey used a convenience sample, we sought to include residents of the hard-hit neighborhoods. A total of 111 participants (86% African American and 14% white) completed the survey, which began with self-administered PTSD screening questions (the civilian version of the screening used in the US military). A total of 52% screened positive for PTSD, compared to US veterans from wars in Iraq or Afghanistan, among whom between 10 and 22% screened positive for PTSD. We then asked each respondent how many murder victims they have known personally. The majority knew five or more individuals who had died by homicide. One of the Rochester qualitative study participants also described their struggles with living in violent areas:

… all they do is kill each other out here. … they just killed that cop. … then another guy was shooting on the corner where I live. … they just constantly killing each other.

*Community level—food deserts*

We conducted an analysis of the availability of healthy food in Syracuse at the request of community members, who bemoaned the lack of grocery markets in their neighborhoods, and said that the small corner stores mainly sold non-food items (e.g., lottery tickets, malt liquor, and mentholated cigarettes). We conducted a logistic regression analysis of the birth weight of infants born to persons living in a census tract with or without a full-service grocery market during their pregnancy (Lane et al., 2008). To control for the potential bias of including premature infants (i.e., those with gestational age of less than 37 weeks), we used full-term IUGR as the outcome. To create the IUGR variable, we removed from the analysis infants born at less than 37 completed weeks of gestation. We then compared those infants (all 37 weeks gestation or greater) who were either low birth weight (less than 2500 grams) or not low birth weight (greater than or equal to 2500 grams). For the risk-factor variable, we used living in a census tract with or without access to a full-service grocery market. We used a secondary database of all births (n = 4506) in the city of Syracuse (2000–2001). Controlling for race and Medicaid, infants of birthing persons residing in a non-supermarket census tract were *three and one-third times* as likely to have full-term unexplained IUGR, compared with infants of mothers living in a supermarket census tract.

This study also evaluated the items for sale at corner markets and convenience stores, which substantiated the community members’ allegations that most sales were of non-food items and that the food sold was often past its expiration dates. We also obtained lottery ticket sales data for Syracuse (2000–2002) and compared the per capita ticket sales in each census tract with the median family income (Lane, 2008). Families with lower incomes bought the most lottery tickets, and in census tracts with higher lottery sales, more infants were born with IUGR.

In the words of one mother who lived in the lowest-income census tract,

“… sometimes [I go to] corner stores because … they be closer than [the grocery stores] … and that’s mostly where my food stamps go to ’cause they’re expensive and closer than everywhere else. I have to take all my kids and walk them to the grocery store and then carry the bags back and it’s so difficult to have to carry bags back with all those kids”.

*Refugee trauma*

Our work with refugees has uncovered the unspeakable tragedies that many suffered prior to being resettled in Syracuse. One birthing individual from Sudan was terrified of giving birth in a modern, high-tech hospital (Lane, 2008). Having arrived already pregnant, she did not understand the various machines and tubes that her obstetricians connected her to when her fetus became distressed. One of her physicians spoke Arabic and the obstetrics team also used the hospital translation service, but it was clear that she was overwhelmed. Fortunately, her infant was born healthy. Part of why she was so fearful was that she had been present when her sister died during childbirth in Sudan. Despite the horrific trauma the refugees face before arriving in Syracuse, they scored as less depressed during pregnancy on the Edinburgh Postnatal Depression scale than US-born persons and had fewer low birth weight births. This paradoxical outcome is called the Migrant Health Effect.

*Composite Case Study*

The composite case study below, drawn from the Rochester qualitative study, illustrates the three levels of trauma described above.

Josefina is a 27-year-old Latina mother of two children, Bianca, age 7, and Miguel, age 6. She reports that each of her pregnancies was challenging but that she received care at the neighborhood health center that had treated her and her family since she was a child. She recounts, “I just had Bianca and then got pregnant with Miguel. During one of my visits to the health center [after giving birth to Bianca], they told me I had post-partum [depression]. I didn’t want nothing to do with her”. Shortly afterward, she found herself pregnant with Miguel. Despite her partner wanting her to have an abortion, she decided to keep the pregnancy.

While she was pregnant with Miguel, she went to a routine prenatal visit. She states, “I went for an ultrasound. The [amniotic] fluids was leaking. They told me, ‘We gotta get him out.’” Realizing that her fetus was unstable, her health providers needed to induce the birth. Josefina states that after Miguel was born, he was transferred to the NICU. Once she was discharged to home, Josefina says her depression worsened. Although she was connected with various services, Josefina reports she often found them unhelpful stating, “I’m not really that fond of some of the nurses, or the social worker or the pediatrician”. Instead of taking time with her, the pediatrician prescribed various antidepressant medications, which she says “…don’t really help me. The medicine makes me feel crazier. So, I just stopped [taking them]”. She states she later realized she needed to focus on her children’s needs claiming that she asked herself, “What is wrong with me?” This realization changed her perception. She said, “I started doing everything for the kids. I wanted to hold them all the time. I looked at them, I played with them, I did everything in my power to make up for all that time”. Her relationship with her partner, however, deteriorated. She states he isolated her from others, reporting, “…he don’t like me to have friends. He don’t like me to talk to nobody, go nowhere. He wants me to stay in the house all the time, cook, clean, and do laundry”.

Josefina also reported the neighborhood crime makes her afraid to leave her home and worried for her children. She states, “The house across the street got people selling drugs and so do the people next door. I’ve seen people threatened with guns. I’ve lived there for two years. I’ve heard at least 80 gunshots over there. If anything happens, we’re in the crossfire. I tell my kids to ‘stay in the front yard.’ So, I keep them in the house”.

Not only is her neighborhood crime-ridden, but she also describes how difficult it is to get healthy food, stating, “My job (at the car wash) doesn’t pay enough for me to afford to get real healthy food for [Bianca and Miguel]”. Due to the high cost of rent and her limited income, Josefina sold her car, which makes it difficult for her to run errands and buy groceries. She states …“I don’t got anyplace I can go to get stuff to make a salad. Even frozen vegetables are hard to find. These little stores don’t have enough room …, but they got plenty of space to sell beer and stuff like that”. In the end she says, “all I can do is my best and try hard to make it”.

## 5. Discussion

Using Bronfenbrenner’s ecological systems theory (Bronfenbrenner, 1983; 1994) we have demonstrated the impact of community-based trauma on low-income birthing individuals, children, and families living in Rochester and Syracuse, New York. The findings demonstrate how trauma does not exist in isolation within intimate partnerships but also within the context of communities and is exacerbated by social policies, institutions, and practices that disproportionately affect marginalized communities.

As in many low-income communities, inner-city neighborhood residents in Rochester and Syracuse face daily stressors, including disproportionate rates of incarceration, poverty, neighborhood violence, IPV, and food deserts that lead to poor health outcomes such as PTSD, depression, IUGR, and IPV and other health issues. These factors often create a cycle of deprivation that is very difficult to escape and perpetuates intergenerational poverty. Laws such as the Rockefeller Drug Law have had a very detrimental impact on African American families including the high incarceration rate among African American males, which in turn has led to female-headed households, greater poverty, and elevated post neonatal mortality in the infants of those families.

Trauma encompasses both physical and psychological dimensions. At the individual level, IPV occurring during pregnancy is associated with severe physical injuries and adverse pregnancy outcomes such as placental abruption. This highlights the urgent need for IPV to be addressed as a public health issue, with a focus on low-income communities where adequate resources for victims are not always present. Social isolation is also a source of trauma resulting from living in high-crime neighborhoods perpetuated by limited available social support systems, as described by Durkheim’s concept of anomie. Following Durkheim, Besnard argued that in such communities, the breakdown of social systems leads to isolation marked by feelings of hopelessness and worthlessness (Besnard, 1988). Social isolation is especially detrimental to new mothers with postpartum depression who lack the support needed to navigate the challenges of early parenthood. Brandt et al. (Brandt et al., 2022) conclude that stress associated with social isolation is associated with worsened addictive, psychotic, and affective disorders while Khoury et al. (2021) found that social support helped protect pregnant persons from depression during pregnancy.

Community-level trauma is characterized by exposure to violence, food insecurity, and the stress that results from living in neighborhoods plagued by gun violence and other forms of crime. Residents reporting high exposure to gunshots and murders leads to widespread fear and symptoms of PTSD. The presence of food deserts further exacerbates health disparities and contributes to poor nutrition and its associated health consequences including IUGR and low birth weight.

Because of the negative health outcomes associated with living in these communities, birthing persons and children require comprehensive interventions that address the social determinants of health including access to healthcare, an enhancement of the neighborhood environment, jobs that pay livable wages, and opportunities to interact and have healthy and fulfilling relationships with family and friends. These interventions need to address all levels of care. At the individual level, targeted mental health services that address the specific challenges low-income birthing individuals face, including postpartum depression and IPV, are needed. At the family level, interventions can focus on supporting single- and two-parent households by addressing the economic and social challenges faced due to low incomes, including advocating for policies that develop educational and job opportunities. At the community level, interventions should tackle the broad structural issues that perpetuate violence and poverty. Advocacy is needed for policy changes that dismantle racial bias in criminal justice, improve access to healthy food by eliminating food deserts, and reduce the prevalence of gun violence. Additionally, at the societal level, comprehensive and culturally relevant healthcare services that all residents have access to, regardless of socioeconomic status, are necessary.

## 6. Conclusions

For more than 20 years, our research team has investigated various harms that cause trauma to people living in marginalized neighborhoods in Rochester and Syracuse, NY, including poverty, IUGR, IPV, a lack of healthy retail food, and social isolation. We have argued that changes in social policies and services need to be made so that interventions can be rendered at all social-ecological levels as put forward by Bronfenbrenner including the individual, family/group, community, and society. Using Bronfenbrenner’s social-ecological theory helps to frame these harms and can serve as a theory base from which to assess interventions so that neighborhoods and communities can function at a higher level, and access to goods and services is equal for all.

## Figures and Tables

**Table 1:** Studies used in this analysis.

Number	Study Topic	Methods, Number of Participants, and Location
1	Study of father involvement at the time of the birth (Keefe et al., 2017).	Conducted in Syracuse. Secondary data analysis of combined births and infant deaths (n = 4343, singleton births, January 2000–March 2002). The database was part of a larger study on “Innovative models to address racial, ethnic, and geographic disparities in maternal and child health”, that was undertaken between 2000 and 2003 and funded by the Health Resources and Services Administration (Lane et al., 2004).
2	Study of intimate partner violence (IPV) among pregnant persons (Leone et al., 2010).	Conducted in Syracuse. Secondary data analysis of combined births and infant deaths (n = 4343, singleton births, January 2000–March 2002).
3	Study of food deserts and IUGR (intrauterine growth restriction) (Lane et al., 2008).	Conducted in Syracuse. Secondary data analysis of combined births and infant deaths (n = 4343, singleton births, January 2000–March 2002), combined with a mapping of the location of full-service grocery markets.
4	Retrospective analysis of all 2010 births in which pregnant individuals received care in a prenatal clinic serving the working poor (Mestad et al., 2016).	Conducted in Syracuse. Researchers reviewed the charts of 245 pregnant persons who screened positive for depression while receiving antenatal care services. They found that greater than one-half of those who screened positive were not adequately referred for care and followed-up on to be certain the care they received was appropriate (data from 2010).
5	Study of gunshots, murders, and community members’ experience of trauma (Lane et al., 2017).	Conducted in Syracuse. Secondary data analysis of gunshots and murders (2009–2015), using a database provided by the Syracuse Police Department. Also, a survey with residents of the neighborhoods with elevated gun violence regarding how many murder victims each knew personally and screening each for post-traumatic stress disorder (PTSD).
6	Interviews with African American and Latinx mothers, who chose to participate because they were experiencing postpartum depression (PPD) and other psychosocial difficulties (Keefe et al., 2021).	Participants (n = 30) scored in the depressed range of the Edinburgh Postnatal Depression Scale and voluntarily participated in one-hour qualitative interviews focused on how they cope while living with PPD. Conducted in a Rochester neighborhood health center and funded by the Fahs-Beck Fund for Research and Experimentation, the New York Community Trust (2013–2014).
7	Comparing refugee births to all other births in Syracuse (Goble et al., 2023).	Conducted in Syracuse. Study of 3 years of Syracuse births (2018, and 2019).

**Table 2:** Level of analysis.

Individual level	Intimate partner violence
Individual level	Social isolation
Family level	Missing men
Family level	Fear of children being killed
Community level	Neighborhood violence and PTSD
Community level	Food deserts
Refugees	Trauma before resettlement

## Data Availability

Data for the study titled “African American and Latina Mothers Living with Postpartum Depression” can be obtained by contacting Robert Keefe. Data for the study “Innovative Models to Analyze and Address Racial, Ethnic, and Geographical Disparities in Maternal and Child Health Outcomes” can be obtained by contacting Sandra Lane.
